# COVID-19 and regional shifts in Swiss retail payments

**DOI:** 10.1186/s41937-020-00061-x

**Published:** 2020-09-25

**Authors:** Sébastien Kraenzlin, Christoph Meyer, Thomas Nellen

**Affiliations:** 1 Swiss National Bank, Banking Operations, Börsenstrasse 15, CH-8022 Zurich, Switzerland; 2 Swiss National Bank, Financial Stability – Oversight, Börsenstrasse 15, CH-8022 Zurich, Switzerland

**Keywords:** COVID-19, Lockdown, Card payment data, Regional sale shifts, E210, E420, E650, E710, R100

## Abstract

This paper analyzes card payments to the retail sector in Switzerland during the COVID-19 crisis. We provide evidence on aggregate effects and regional shifts. Pronounced shifts—which persisted post-lockdown—can be observed from urban to suburban and rural areas and among cantons. Data allow us to identify directly two sources of shifts: “tourists and business travelers” and “e-commerce.” We indirectly identify additional sources: infection risk, lockdown measures, working from home, shopping tourism, and cash substitution. The COVID-19 crisis seems to have reinforced pre-existing trends that may have faster than anticipated effects on the economy. Our analysis underscores the usefulness of real-time card payment data to inform policymakers.

## Introduction

This paper documents the effects of the COVID-19 crisis on card payments to the retail sector in Switzerland. COVID-19 and related lockdown measures contributed to a substantial drop in such “retail payments.” Even before major lockdown, measures were lifted, retail payments started to rebound quickly, and soon rose far above pre-lockdown levels. We further provide evidence that regional shifts occurred in retail card payments across areas with different levels of urbanization and across the 26 Swiss cantons. Regional shifts exceed aggregate effects and have remained elevated despite two rounds of lockdown easing and the subsequent general increase in retail card payments.

While aggregate effects are noteworthy and in need of explanation, documented regional shifts hint at structural changes that were accelerated by the outbreak of COVID-19. If regional shifts are here to stay, the economy may face multifaceted effects long after COVID-19 restrictions have ended. To mention just one potential impact, regional shifts may affect the rent and real estate value of retail locations. This raises two questions. First, which sources have contributed to the documented effects, and in particular to the regional shifts? Secondly, are these sources likely to be associated with permanent shifts in retail payments, or will they instead be temporary?

We find direct evidence of shifts being associated with a canton’s dependence on “tourism and business travel” (as measured by payments made with cards issued by foreign financial intermediaries) and with its economy’s “e-commerce” intensity (as measured by cantonal card-not-present payments, i.e., payments at terminals that are associated with e-commerce). Payments made with foreign-issued cards (foreign cards) relate positively to these shifts, i.e., lost card payments from foreign tourists and business travelers increase these shifts. Similarly, card-not-present payments increase such shifts, because the retail sectors of cantons diverge in their e-commerce intensity.

We further provide evidence of sources that we cannot trace directly in the card payment data set, but indirectly through the correlation of proxies with card payments. These sources are the following. First, we consider infection fear, as proxied by a canton’s exposure to COVID-19 infections; second, the retail sector’s exposure to lockdown measures, as measured by the sectoral lockdown index; third, the intensity of “shopping tourism,” as proxied by two novel indices, namely annual shopping tourism frequency and shopping tourism accessibility; forth, the feasibility of working at home, as proxied by the home office index; and fifth, cash substitution as measured by a novel indicator for cash usage, namely the share of cash withdrawals in payments at the point of sale (POS). Shopping tourism’s effect on shifts is ambiguous. All other sources exacerbate shifts.

Three sources might dissolve rather quickly, assuming a second COVID-19 wave is prevented in Switzerland and neighboring countries. Infection fear might ease and people will move back to normal shopping habits to the extent that is feasible. As lockdown measures are eased further, and as people adapt to the new social distancing and hygiene rules, the effects of lockdown measures will at least be substantially reduced. Similarly, shopping tourism has come almost to a complete halt but is expected to recover quickly once borders are open again.

Other sources will continue to contribute partly or fully to these shifts. It remains open as to whether or not tourism and business travel will reach pre-COVID-19 levels, and if so, what the time horizon for this might be. The remaining sources tend to represent secular trends that have been on the radar of the retail sector for some time. We argue that the COVID-19 lockdown has allowed us a glimpse into the crystal ball, offering a natural experiment for what the effects of these trends might look like in the future. Moreover, COVID-19 might prove a catalyst for these trends, making the future arrive faster than anticipated. Shifts related to these sources might thus decrease as life goes back to normal, but are unlikely to be reversed completely.

In particular, we expect e-commerce to keep some of the market share gained during the lockdown and to continue its pre-existing growth path from this elevated level (Christen, Fuhrer, Hotz, & Jucker, 2020). After the experiences gained during the lockdown, work from home is also expected to gain traction. COVID-19-related survey evidence is compatible with the perception that the share of hours spent working at home will increase substantially beyond pre-COVID-19 levels (gfs.bern, 2020). Both Swiss employees and employers perceive working from home as beneficial and are willing to increase its share (Rütti, 2020).

Cash payments make it difficult to translate card payments directly into turnover or consumption. Changes in cash usage exacerbate this problem. While cash substitution in Switzerland was a comparatively slow process before COVID-19 (SNB, 2018), the outbreak of COVID-19 may prove a catalyst towards a more frequent use of non-cash payment instruments in the future. As statistics on cash usage are scarce, we build a new proxy for cash usage (the share of domestic cash withdrawals in payments at the POS). Our proxy indicates an aggregate decline in cash usage. This drop partially reflects growth in card payments. Cash usage also differs among cantons and exacerbates card payment shifts. The SNB’s upcoming Survey on Payment Methods in 2020 will be able to answer the question as to whether a substitution was a transitory effect, or whether COVID-19 has induced a more permanent switch to non-cash payment instruments.

We provide aggregate and regional evidence from a new database on card transactions. We do so based on more than 1.3 billion debit and credit card transactions. Transaction level data are collected from the largest acquirer in Switzerland, which has an approximate share of two thirds in the acquiring business. Acquirer data implies that the perspective of Swiss merchants is taken. We focus on the retail sector, as card transactions in other sectors essentially came to a halt during the COVID-19-related lockdown period from mid-March to the end of April 2020. Other sectors further remained subdued during the two phases of lockdown easing, until the end of May 2020, which also forms the end of our sample.

From a policy perspective, our paper provides an initial insight into the potential of card payment data. On the one hand, data allow us to track the economy in real time on the basis of effective transactions rather than indirect indicators such as Internet searches. On the other hand, these data can also be used to study structural forces that shape the economy. Our analysis thus underscores the usefulness of real-time payment data to inform policymakers.

Section 2 describes the card payment data, the proxy indicators, and the descriptive methodologies applied. Section 3 illustrates the aggregate effects of the lockdown, further breaking down aggregate effects into sectoral effects. Section 4 provides evidence on regional shifts in card payments across different degrees of urbanization and across cantons. Section 5 discusses sources of retail payment shifts that can be traced in the data directly. Section 6 provides indirect evidence on sources of shifts that cannot be directly traced in the data. Section 7 discusses the results in light of the literature and briefly reflects on the potential of card payment data.

## Card payment data, proxy indicators, and methodology

We analyze debit and credit card payments—with a focus on the retail sector—using transaction data from Worldline/Six Payment Services (Worldline/SPS).^1^ Card payments to domestic merchants are available at the transaction level. The granular transaction-level data can be analyzed according to the following criteria: merchant category according to the general classification of economic activities (NOGA) at the two-digit level (NOGA-2),^2^ the degree of urbanization of a merchant’s municipality,^3^ the canton of the merchant,^4^ card-present transactions (payment at POS) or card-not-present transactions (e-commerce), and the country of the (domestic or foreign) card issuer.^5^ Further details on sources and the construction of data are explained in Appendix 1.

In terms of card transactions conducted in Switzerland, the data are likely to be representative, covering roughly two thirds of card transactions conducted in Switzerland and roughly 40% of all retail payments.^6^ Non-card retail payments are either settled in cash, via mobile payments, or via invoices. In 2017, 45% of the value of consumer payments was still settled in cash (SNB, 2018). A look at the share of cash withdrawals in POS payments and cash withdrawals indicates that cash usage has declined and that card payments have gained additional traction since the lockdown (see Fig. 4 for the share of cash withdrawals in payments at the POS and cash withdrawals and Brown, Fengler, Lalive, & Rohrkemper, 2020, for a similar analysis). Granular data for mobile payments are not available. Most mobile payment services are settled via card payments, but Twint, Switzerland’s largest mobile payment service provider, often directly connects to customers’ sight deposits at their respective banks. While Twint is said to have gained value, volume, and users since the outbreak of COVID-19, its share in the value of total payments probably remains comparably small (SNB, 2018). Similarly, invoices and other forms of payment constitute a negligible share of retail payments in particular (SNB, 2018).

**Table 1:** Correlations of excess domestic card-present retail payments with selected indicators across the pre-lockdown, lockdown, and post-lockdown periods. Source: Own calculations, Worldline/SPS, SIX BBS AG, SFSO

	January/February weeks		Lockdown weeks		April/May weeks	
	Correlation	*p* value	Correlation	*p* value	Correlation	*p* value
**All 26 cantons**						
Lockdown index retail sector	− 0.38	0	− 0.49	0	− 0.33	0
New COVID-19 cases per 100,000 inhabitants	NA	NA	− 0.50	0	− 0.20	0.02
Home office index, unadj.	− 0.45	0	− 0.51	0	− 0.74	0
Cross-border shopping index, frequency	− 0.09	0.29	− 0.25	0	− 0.20	0.02
Cash usage, change over period	NA	NA	− 0.65	0	− 0.28	0.01
**Excluding eight cantons with high COVID-19 infection rates**						
Lockdown index retail sector	− 0.53	0	− 0.51	0	− 0.43	0
New COVID-19 cases per 100,000 inhabitants	NA	NA	0.02	0.86	0.07	0.53
Home office index, unadj.	− 0.62	0	− 0.44	0	− 0.72	0
Cross-border shopping index, frequency	− 0.31	0	0.11	0.31	0.33	0
Cash usage, change over period	NA	NA	− 0.57	0	− 0.46	0

We rely on data from January 2019 to May 31, 2020, covering more than 1.3 billion card transactions. We divide the sample into three periods, the *pre-lockdown* period (Tuesday, January 7, to Monday, February 17), the *lockdown* period (Tuesday, March 17, to Sunday, April 26), and the *post-lockdown* period (Monday, April 27, to Sunday, May 31). We restrict the pre-lockdown period to Monday, February 17, in order to avoid pre-lockdown effects in response to the outbreak of COVID-19; this first period serves as the pre-COVID-19 benchmark period.

While China already implemented lockdown measures in January, the first European COVID-19 infections were reported in Italy on January 31. The first Swiss case dates from February 25 and was followed by the Swiss Confederation’s ban on public events exceeding 1000 participants on February 28 and its public campaign on hygiene protection measures on March 1. On March 11, the WHO declared a worldwide pandemic and the Canton of Ticino declared a lockdown, followed by the Confederation on March 16.^7^ The post-lockdown period starts with the first easing of the lockdown in Switzerland on Monday, April 27, 2020, and includes the second lockdown easing on Monday, May 11. On Monday, April 27, shops were partially reopened, and on Monday, May 11, shops were completely reopened.^8^

We calculate the variable *excess total*, *retail*, and *non-retail card payments* for values and volumes. These variables are calculated as the percentage differences between payments for specific periods (days, weeks, or periods as defined above) in 2020 and 2019. In line with others (Andersen, Hansen, Johannesen, & Sheridan, 2020; Carvalho et al., 2020), we match weekdays to construct periods. This is necessary due to noticeable weekday patterns found in card payment data (Brown et al., 2020). When analyzing weekly data, we center weeks around Tuesdays and start with Tuesday, March 17, 2020, the first day of the lockdown. In other words, we compare Tuesday, March 17, 2020, to the 364th day before, i.e., Tuesday, March 19, 2019.^9^ We further smooth weekday patterns that may deviate before and after COVID-19 by calculating and comparing weekly averages. For instance, the first week of the lockdown period started on Tuesday, March 17, and ended on Monday, March 23, 2020, which is then compared to the week starting Tuesday, March 19, 2019, and ending Monday, March 26, 2019.^10^

While retail payments show day-of-the-week seasonality, our weekly comparisons and the comparison over the lockdown period match very similar weeks and time periods. Furthermore, payday effects are much less pronounced for debit and credit card payments as compared to cash withdrawals (Brown et al., 2020). Considering the 25th of each month as the most regular payday date in Switzerland, payday effects mostly occur within matched weeks, which mitigates potential distortions.

We consider seven sources of retail payment shifts across areas with different degrees of urbanization and across cantons. The first two sources can be directly identified in our data set via the surgical extraction of respective payments.

1. The first source is related to *foreign tourism and business travel*. We identify payments associated with foreign tourism and business travel by extracting payments with foreign cards. We believe that the absence of travel exacerbates shifts, as cities and specific tourist hot spots in rural areas are prime travel destinations.
2. *E-commerce* is the second source. We identify e-commerce payments by extracting card-not-present payments after having extracted foreign card payments, i.e., we focus exclusively on domestic e-commerce. As e-commerce intensity is unlikely to be distributed equally among different regions, we believe that e-commerce exacerbates shifts.

We evaluate the impact of the remaining five sources by looking at the correlation of their proxy indicators with the calculated variable excess domestic card-present payments per canton. In Appendix 2, we consider alternative proxy indicators as robustness checks.

3) A stronger cantonal exposure to COVID-19 may have subdued domestic card-present retail payments due to *infection fear*. People avoid public places and reduce their consumption of basic goods and services even further than had been made inevitable by the imposition of lockdown measures. We consider the COVID-19 infection cases per 100,000 residents as an overall indicator of a canton’s COVID-19 exposure. To proxy infection fear, we consider *growth in infection cases*, i.e., we take the difference of cantonal end-of-period infection cases for this and the previous period. Note that these values are zero for the pre-lockdown period. When we consider infection fear as a source, we must note that particularly vulnerable persons might have reduced their consumption even more extensively. We therefore consider the share of residents above the age of 65 as an alternative proxy indicator.
4) The retail sector in different cantons may be more or less strongly hit by the lockdown and by measures such as social distancing and hygiene rules. The sectoral lockdown index by Faber, Ghisletta, and Schmidheiny (2020) measures these frictions and can be applied to the retail sector per canton. As essential sectors remained open during the lockdown period, we use the share of the non-essential retail sector’s labor force in the whole retail sector’s labor force as an alternative proxy indicator.
5) We proxy a canton’s work from home relying on the *home office index* per canton by Faber et al. (2020). The home office index measures the regional economy’s potential to perform jobs from home. Regions hosting more urban areas and attracting employees from outside the region usually have a larger share of jobs that can be performed from home and will likely lose domestic card-present retail payments relative to regions that have lower home office index values. When analyzing cantonal shifts, the natural robustness check is to correlate excess retail card payments with the proxy indicators of commuting intensity and the cantonal degree of urbanization. The cantonal degree of urbanization is defined as the share of residents living in urban municipalities.
6) Shopping tourism by Swiss residents is popular due to substantial gains in purchasing power and deductible value-added tax. However, shopping tourism was prohibited during the lockdown and the two subsequent easing phases (at least for POS shopping, i.e., for card-present payments). Consequently, we expect cantons with a relatively larger intensity of shopping tourism to have larger excess retail payments after the lockdown. Due to a lack of data on shopping tourism, we constructed an annual proxy indicator for *shopping tourism frequency* per capita together with Demo SCOPE AG and a proxy indicator for *shopping tourism accessibility* per canton together with BAK Economics AG (see Appendix 1 for details). We use the shopping tourism frequency indicator in the main text. The shopping tourism accessibility indicator is used in Appendix 2.
7) Consumers may have avoided cash if they perceived it as a potential virus carrier since the outbreak of COVID-19 and so resorted to card payments.^11^ While reduced *cash usage* is a secular trend, we simply look at cash usage from the perspective of aggregate excess card payments and payment shifts. We proxy cash usage, considering the share of cash withdrawals in POS retail payments and cash withdrawals conducted with domestic cards (see Appendix 1). The pre-lockdown period serves as the benchmark, as we set the change in cash usage as equal to the difference between periods. Consequently, pre-lockdown period values are zero by construction.

## Aggregate effects

**Fig. 1:**
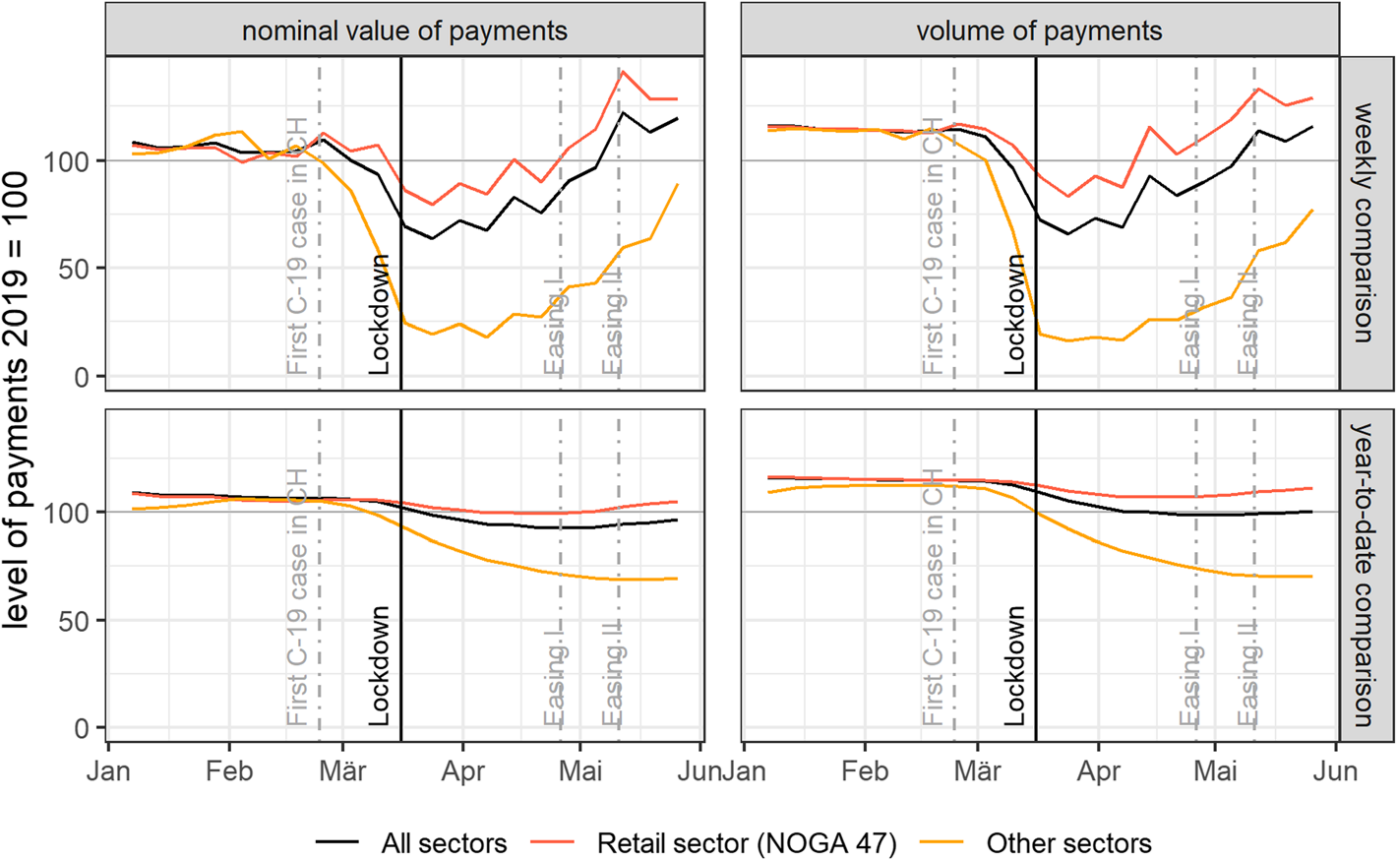
Card payment activities in Switzerland in 2020. Source: Own calculations, Worldline/SPS

We start by providing evidence on *aggregate* payment effects, looking at total card payments, retail card payments (NOGA-47 class), and non-retail card payments (all other NOGA classes). The upper panel in Fig. 1 depicts weekly excess value and excess volume of transactions. Excess values and volumes are represented as 2020 payments relative to their respective 2019 numbers. The lower panel shows accumulated weekly excess numbers.

The upper panel in Fig. 1 indicates a pronounced drop in total debit and credit card payments in line with the findings of other literature and—in a Swiss context—specifically in line with Brown et al. (2020). The drop in retail card payments is less pronounced than the sharp drop in non-retail card payments. The lower panel in Fig. 1 depicts the accumulated excess card payments from the beginning of the year. While growth in card payments explains part of the dampened effects in comparison with the weekly excess card payments in the upper panel, this figure points to the structural changes that dampen the effects on card payments.

Excess retail payments behaved normally in January and February, being slightly above zero and thus reflecting growth in card usage and consumption. This trend reversed several weeks before the lockdown became effective, at around the time when the first COVID-19 infection was confirmed in Switzerland. In terms of value and volume, we find that excess retail (non-retail) card payments experienced a sharp decline during the lockdown period, bottoming out at around − 20% (− 80%) before recovering to varying degrees by the end of our sample, with year-on-year figures of around + 40% (− 20%). These developments are unprecedented and deserve an explanation.

**Fig. 2:**
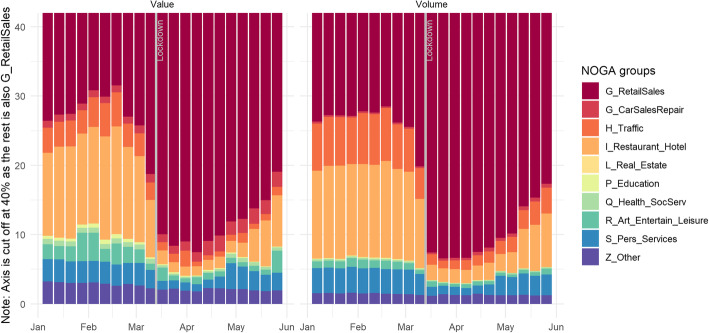
Shares of the NOGA 2 groups for value and volume in total card payments, measured by weekly averages for the period January to May 2020. Source: Own calculations, Worldline/SPS

Let us start with a closer look at sectoral differences in the drop in excess card payments. This is less surprising given the lockdown measures, i.e., the closure of all shops except those for essential goods and services. Looking at the sectoral breakdown in Fig. 2 that displays the share of NOGA-2 groups for value and volume of card payments, this is well-reflected. All but the retail sector almost ceased to exist during the lockdown period, with total card payments in non-retail sectors falling to levels as low as 10% in a year-on-year comparison.

Figure 2 further reveals that the retail sector is dominant within overall card payments, both in terms of value and volume, before, during, and after the lockdown. Furthermore, we find that the lockdown hit the retail sector as a whole less severely, because this sector includes most so-called essential services that were exempt from the closure of shops during the lockdown period. By contrast, the share of card payments in non-retail sectors dropped drastically. The non-retail sectors’ aggregated share amounted to more than 25% pre-lockdown and plummeted during lockdown to below 10%. As shown in Fig. 1, retail card payments remained at a higher level during lockdown.

**Fig. 3:**
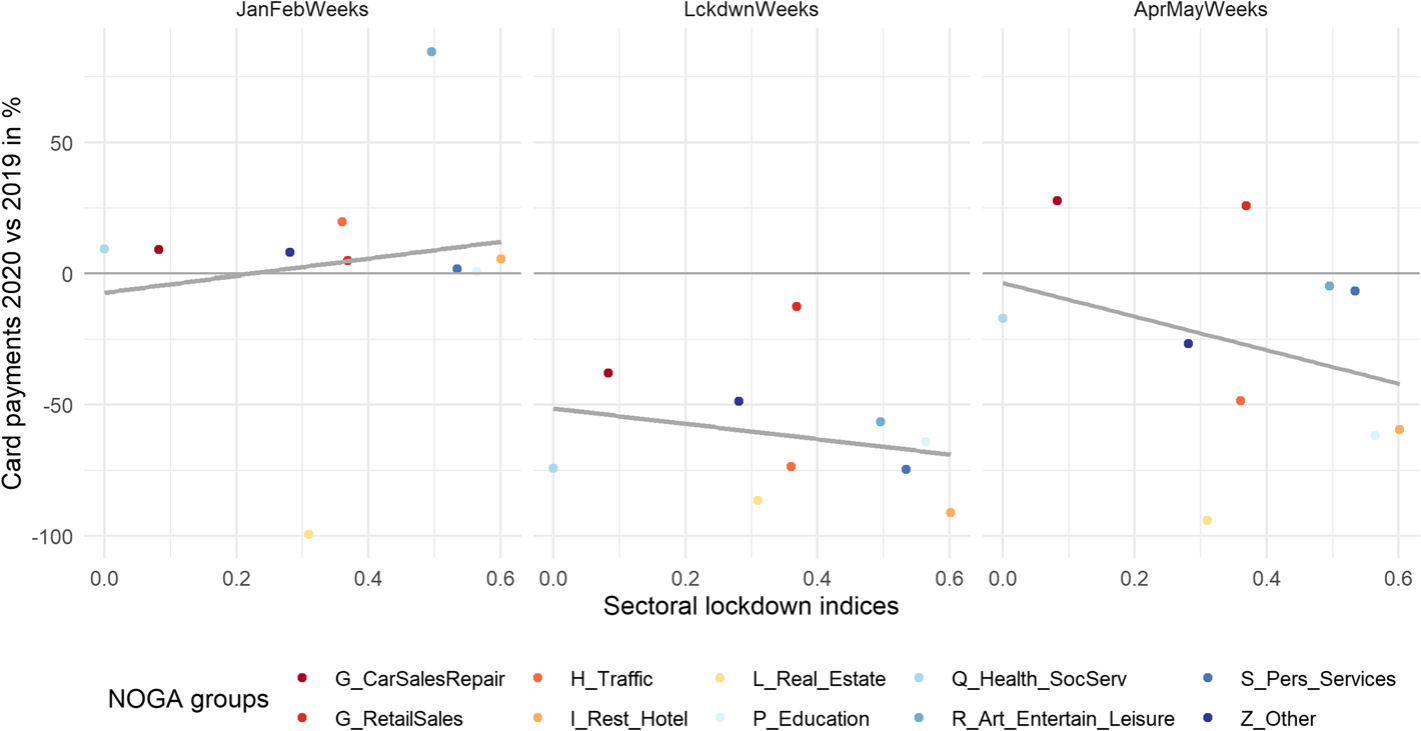
Excess card payments in percent by the NOGA-2 groups versus corresponding “sectoral lockdown indices” for pre-lockdown (left), lockdown (middle), and post-lockdown (right) periods. Source: Own calculations, Worldline/SPS, SFSO

Both retail and non-retail sectors had started to drop before the lockdown became effective, as can be seen in the first row of Fig. 1. While card payments would have been subdued even without the lockdown, the forced closure of shops had a further detrimental effect on excess card payments. This is mirrored in Fig. 3, which displays a scatter plot of NOGA-2 groups’ excess card payments in percentages against these groups’ individual sectoral lockdown indices. Almost no correlation is seen in the pre-COVID-19 period. During the lockdown period, however, the relationship becomes negative and remains clearly negative in the post-lockdown period—despite an initial revival of activity. This is related to the fact that many sectors remained subject to closure and other lockdown measures (such as hygiene rules and social distancing).

It is not surprising that the drop in excess card payments can be attributed to the lockdown and related measures. As shops gradually opened during the post-lockdown period, the recovery of growth in excess card payments is also hardly a surprise. However, as many lockdown measures stayed in force during the post-lockdown period, it is harder to explain the positive excess retail card payments and the generally strong growth in card payments. There are three sources for retail and card payment growth, though these are only indirectly identifiable: cash usage, e-commerce, and shopping tourism.

**Fig. 4:**
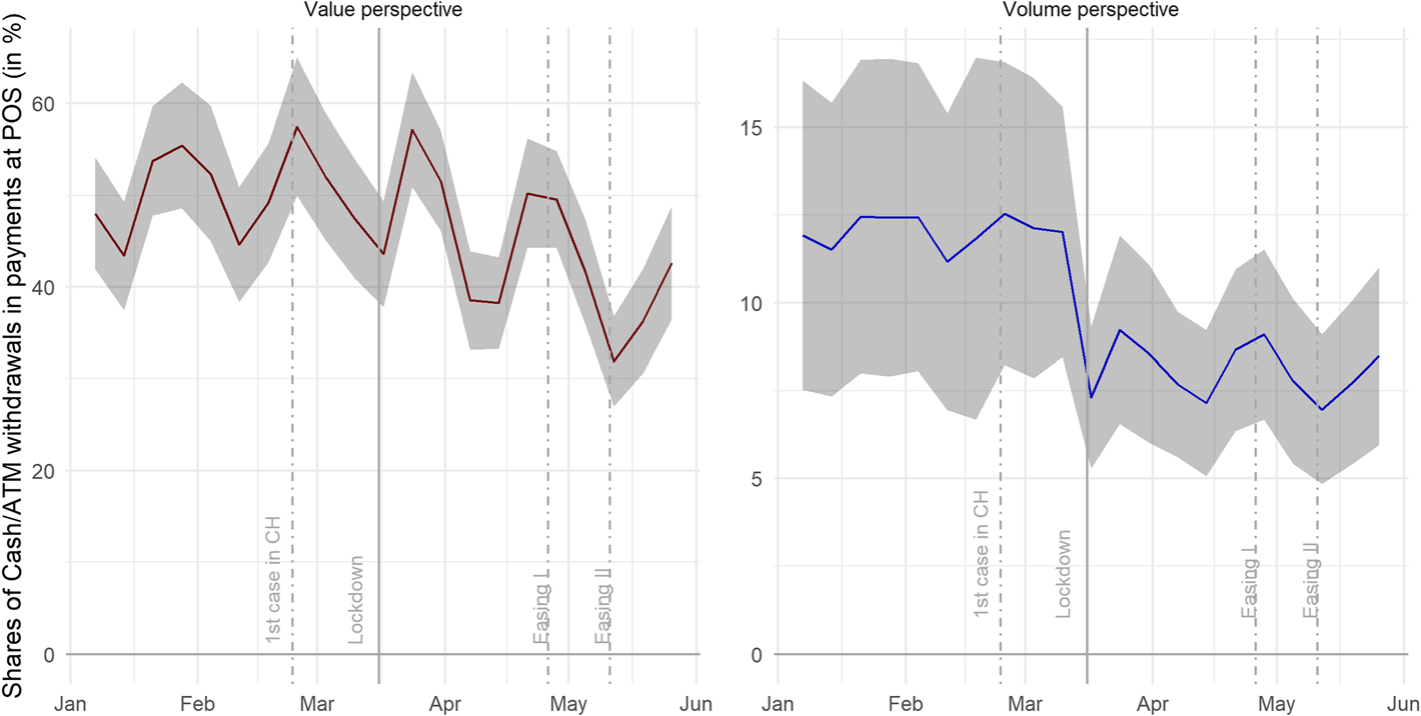
Cash withdrawals relative to payments at POS (domestic card-present retail payments and cash withdrawals), as measured by weekly activities in terms of value (lhs) and volume (rhs) in 2020. Source: Own calculations, SIX BBS AG, Worldline/SPS

First, there is a noticeable decline in cash usage. Figure 4 shows the share of the weekly value of cash withdrawals in the weekly value of POS payments to the retail sector in 2020 (domestic card-present payments and cash withdrawals). It shows the median of cantonal shares together with the interquartile range (shaded area). Two insights can be gained here. First, this indicator fell over the period of investigation, implying that cash payments began losing their market share after the outbreak of COVID-19. While the steady growth of card payments represents a secular trend, the decline of the cash share seems to have gained traction with the outbreak of COVID-19. Secondly, the variation among cantons has approximately halved over time, implying that high cash usage cantons have caught up with card payments.

It is interesting to note that cash usage (see red line in Fig. 4) roughly matches the share of 45% in terms of the value of cash payments found in the most recent payment method survey by the SNB (SNB, 2018). The noticeable peak-trough pattern is related to weeks with salary payment dates. After receiving their salaries, many Swiss residents withdraw cash that is partly used to pay invoices at counters of financial intermediaries. The low values during January are due to end-of-year and Christmas effects that leave Swiss residents cash-rich in January and February. This effect leads to an underestimation of the cash usage at the beginning of the year and, consequently, to an underestimation of the substitution that takes place afterwards.

**Fig. 5:**
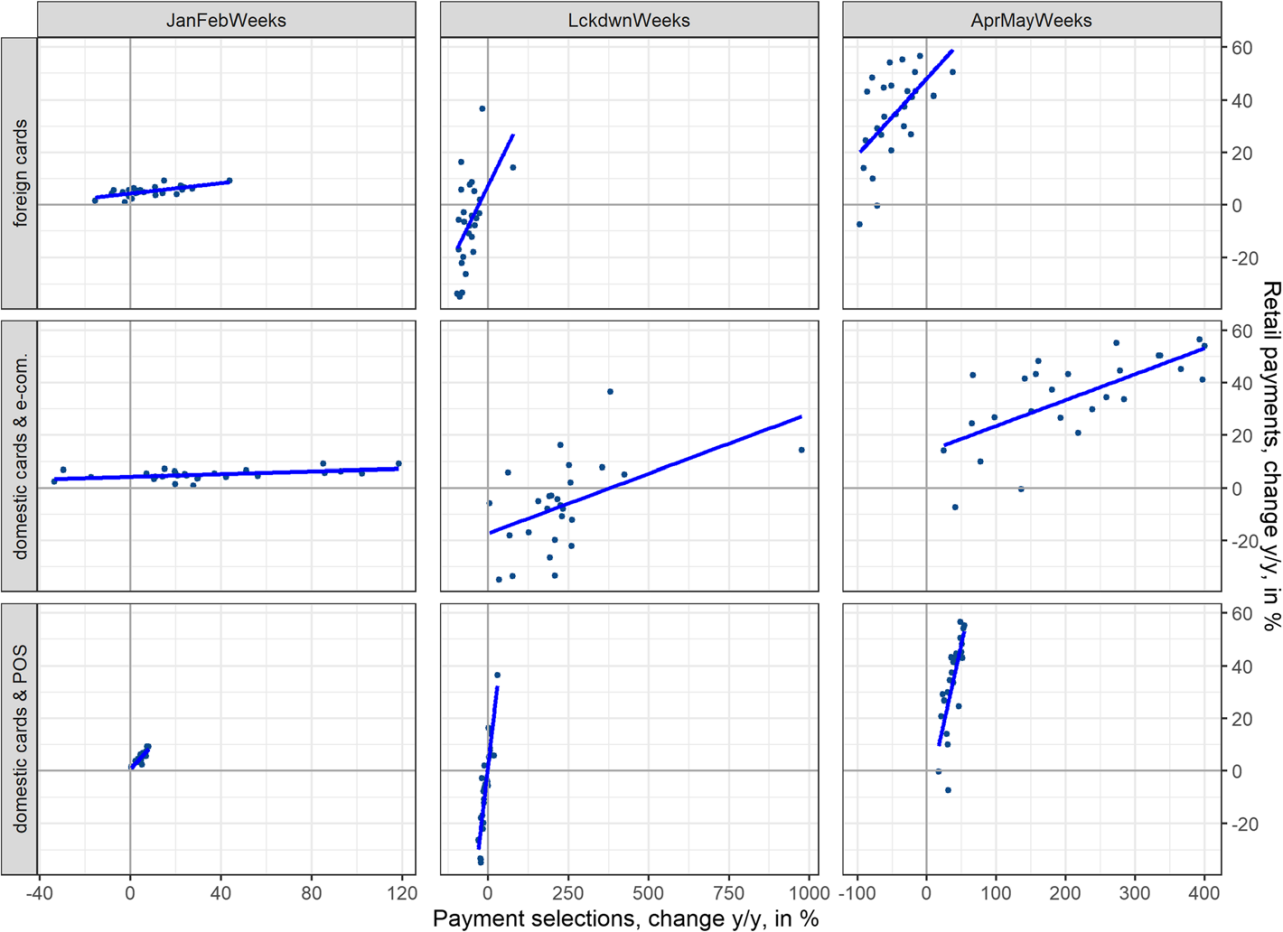
Excess retail card payments (percentages) for foreign issued cards, domestic card-not-present payments, and domestic card-present payments (panels) versus total retail payments (percentages) per canton for the pre-lockdown (left), lockdown (middle), and post-lockdown (right) periods. Source: Own calculations, Worldline/SPS

Moreover, it is important to note that our cash usage indicator uses domestic card-present retail payments in the denominator. The idea is to build an indicator with close substitutes only. The drawback is that this cash usage indicator does not account for substitution effects between POS and e-commerce sales.^12^ The decline in cash usage is thus stronger than indicated by our proxy indicator. This is important to acknowledge, as e-commerce has thrived since the outbreak of COVID-19 (see the third column of Fig. 5).

However, the lockdown appears to have hit sectors with a larger cash share (bars and restaurants, for instance) relatively more strongly than sectors with a lower cash share. Also, other forms of cash use may have been subdued, such as person-to-person payments (e.g., gifts). Such sources may show a rebound in cash usage after further easing steps.

Thirdly, cross-border shopping tourism remained prohibited from the lockdown until mid-June. This likely resulted in markedly higher excess retail payments in cantons with high shopping tourism exposure. We will come back to this topic when analyzing regional shifts. A quantitative analysis of the extent of cross-border shopping tourism based on aggregated issuer data can be found in Brown et al. (2020). The evidence presented by Brown et al. (2020) shows that shopping tourism attracts non-negligible payment values.

## Retail payment shifts

Figures 1 and 2 reveal that the retail sector is dominant in total card payments, both in terms of value and volume. Also, economic activity in the retail sector remained substantial during the lockdown. We therefore focus on retail card payments when analyzing regional shifts. We first analyze shifts across the 26 cantons and then discuss shifts across areas with varying degrees of urbanization, as defined by the SFSO’s spatial classification “Raumgliederungen.”

**Fig. 6:**
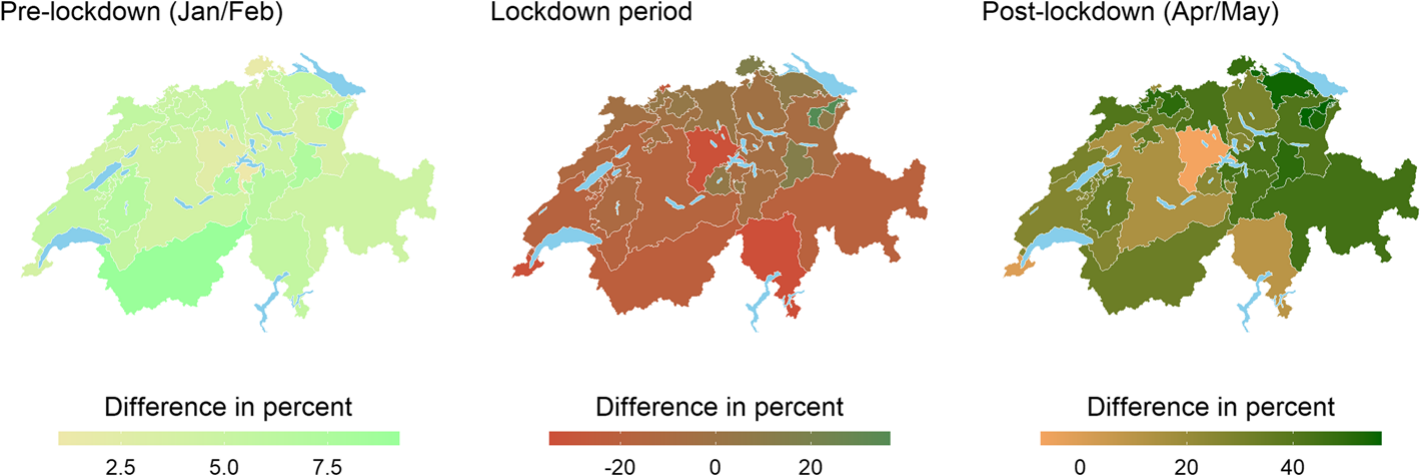
Average excess retail card payments (percentages) per canton for pre-lockdown (left), lockdown (middle), and post-lockdown (right) periods. Source: Own calculations, Worldline/SPS, SFSO

Figure 6 provides evidence on retail payment shifts across cantons. Excess retail payments varied between 0 and 7.5% before the lockdown. These numbers changed to − 30% and + 30% during the lockdown period, implying a substantially elevated heterogeneity. Furthermore, excess retail payment shifts remained at elevated levels during the post-lockdown period, varying between − 10 and + 50%. We validate this evidence on regional shifts by means of standard heterogeneity measures in Appendix 2.

**Fig. 7:**
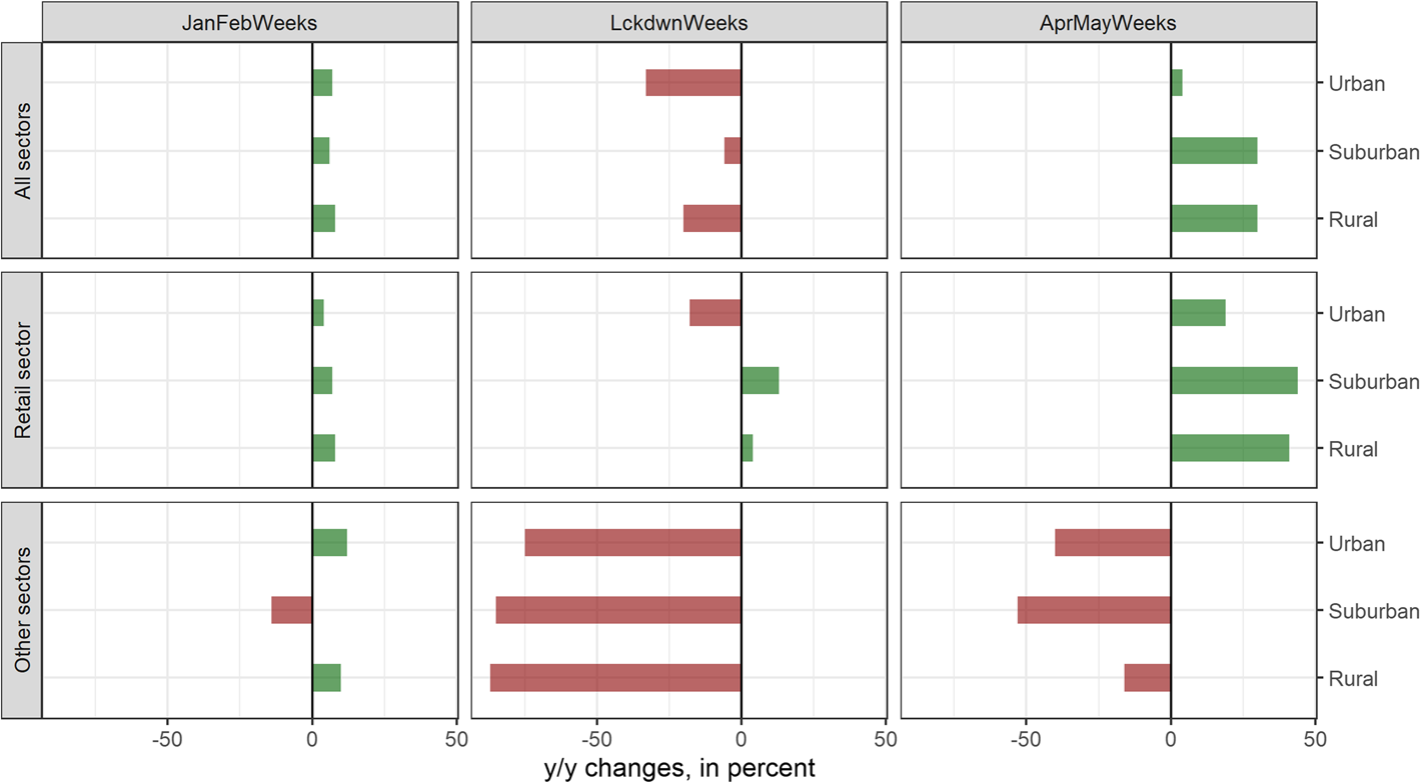
Excess card payments (percentages) for all sectors (upper panel), the retail sector (middle panel), and all other sectors (lower panel), differentiated by urban, suburban, and rural areas in Switzerland (sub-panels) in the pre-lockdown (right), lockdown (middle), and post-lockdown (left) periods. Source: Own calculations, Worldline/SPS

Figure 7 depicts the shifts of card payments across areas with different levels of urbanization. Similar to the total excess card payments (for retail payments and all other sectors), excess card payments across different levels of urbanization were slightly above zero and fairly homogenous during the pre-lockdown period, reflecting growth in card usage and consumption. During the lockdown, excess card payments diverged to a much greater extent. Urban areas witnessed excess retail payments of − 18%, whereas excess retail card payments in suburban and rural areas amounted to + 13% and + 4% during the same period (middle panel, middle graph). During the post-lockdown period, recovery in retail card payments remained subdued in urban areas compared to suburban and rural areas (middle panel, graph on the right-hand side). This implies that shifts remained at an elevated level. Due to the dominance of the retail sector, the overall picture does not look much different (upper panel, all graphs) from the picture for retail payments (middle panel).

## Directly identifiable sources of regional shifts

The available retail card payment data directly contains two potential sources of regional shifts. First, shifts may result from the absence of foreign tourists and business travelers. Secondly, shifts may be caused by regionally diverging e-commerce intensities.

**Fig. 8:**
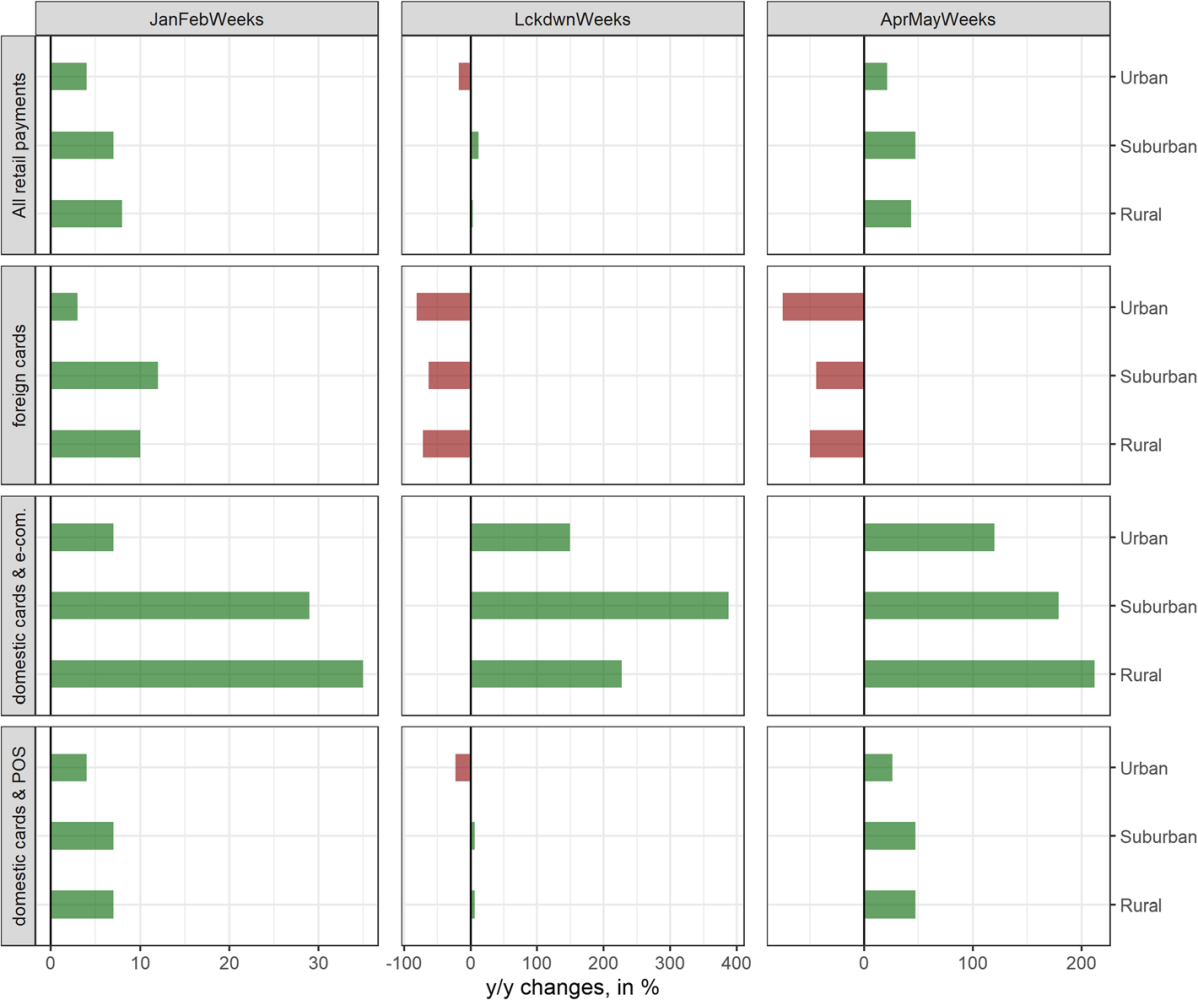
Excess retail card payments (percentages) differentiated according to all payments (first panel), foreign-issued card payments (second panel), domestic card-not-present payments (third panel), and domestic card-present payments (fourth panel), and differentiated by urban, suburban, and rural areas (sub-panels) for the pre-lockdown (left), lockdown (middle), and post-lockdown (right) periods. Source: Own calculations, Worldline/SPS

We first analyze the contribution of foreign tourists and business travelers to retail card payment shifts. Figure 8 depicts the regional heterogeneity in terms of urbanization. Urban and rural areas suffer more from the absence of foreign tourists and business travelers, with urban areas suffering most of all. As the contribution of these payments to total retail card payments is non-negligible (almost 10% in 2019), the absence of tourists and business travelers contributes to the heterogeneity among areas with differing degrees of urbanization. The absence of foreign tourists and business travelers also contributes to shifts in retail payments across cantons. Figure 5 (first panel) shows the excess foreign-issued card retail payments versus excess total retail card payments per canton. The positive correlation indicates a rather strong contribution to retail payment shifts across cantons.

Note that the acquirer nature of our data does not allow us to account for e-commerce purchases by domestic residents abroad. Given the increase in domestic e-commerce activities, e-commerce purchases abroad might have increased too. In terms of retail card payments, it is unclear whether this increased or decreased regional shifts related to e-commerce.

We now move on to domestic card-not-present retail payments that essentially represent e-commerce-related retail payments. E-commerce had been growing steadily, even before COVID-19, but has gained considerable traction during the crisis and has therefore contributed significantly to the cantonal shifts. After the lockdown until the end of May, domestic card-not-present payments accounted for almost 13% of total card retail payments. In the same period in 2019, the share of domestic card-not-present payments in total card retail payments had been around 6%, i.e., less than half of the 2020 share. Looking at Fig. 8 (third panel) and Fig. 5 (middle panel), shifts in total retail card payments are positively influenced by card-not-present payments across differently urbanized areas and across cantons. While excess retail card-not-present payments decreased in the post-lockdown period, a positive contribution to shifts across differently urbanized areas remains and strengthens across cantons.

## Indirectly identifiable sources of regional shifts

In what follows, we evaluate sources of regional shifts that cannot be traced directly in the data. This implies that we cannot study their effects on shifts between areas with different degrees of urbanization. However, we can do so for shifts among cantons.

**Fig. 9:**
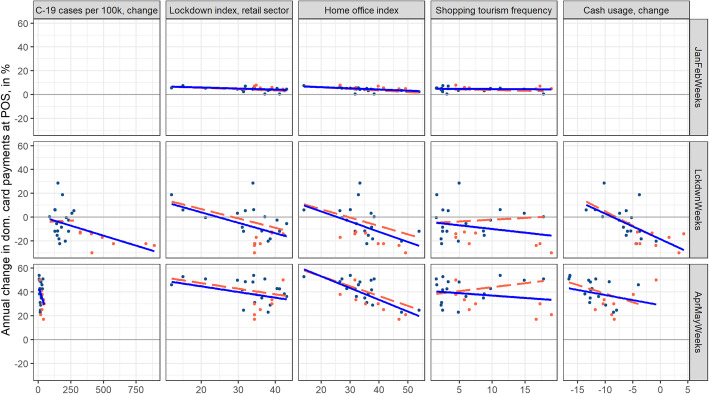
Excess domestic card-present retail payments versus further sources of shifts per canton for pre-lockdown (upper panel), lockdown (middle panel), and post-lockdown periods (lower panel). Source: Own calculations, Worldline/SPS, SFSO

**Fig. 10:**
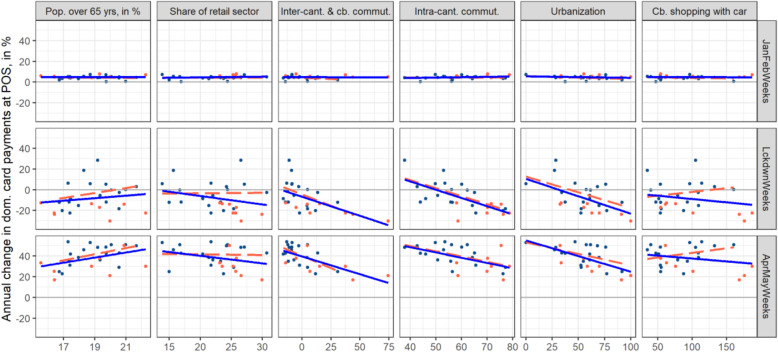
Excess domestic card-present retail payments versus further sources of shifts per canton. Source: Own calculations, Worldline/SPS, SFSO

Next to the declining use of cash, we believe four additional sources to be relevant for payment shifts: infection fear, differences in retail-sector lockdown exposure, differences in the feasibility of working from home, and differences in shopping tourism exposure. Due to the low number of 26 cantons and the static nature of most indicators, we simply look at the indicators’ correlation with excess domestic card-present retail payments for the three different periods under consideration. Table 1 in Appendix B.2 depicts these correlation statistics. As a robustness check related to all sources, we display trend lines in Figs. 9 and 10 in Appendix B.3 both in terms of all cantons (blue) and only for those cantons with an infection rate below 300 infections per 100,000 residents (red). As a further robustness check, we evaluate correlations of alternative proxies meant to capture the same effect (Figure B.3 and Table 2 in Appendix B.3). Restricting the dataset to domestic card-present payments allows us to focus on domestic differences (infection fear, lockdown exposure, home office feasibility, shopping tourism exposure, cash usage). Furthermore, domestic card-present payments represent the most likely substitutes for cash payments and shopping tourism.

Figure 8 in the previous section shows regional shifts. These remain notably strong, amounting to almost 25 percentage points of inter-regional differences in excess domestic card-present payments for the lockdown and post-lockdown periods (as compared to below 3 percentage points during the pre-lockdown period). Figure 9 displays the respective scatter plots that show cantonal shifts in response to the five sources mentioned.

Not all cantons were hit in the same way by COVID-19. Southwestern cantons witnessed higher infection rates than the other cantons. Urban cantons also witnessed higher infection rates than rural cantons. We proxy infection fear by end-of-period growth in infection rates per 100,000 residents. During the pre-lockdown period, no COVID-19 infection was recorded (the first graph in the first column of Fig. 9). By the end of the lockdown period, COVID-19 infection rates had grown exponentially, and with them, payment shifts among cantons (the second graph in the first column). The blue trend line visualizes the correlation among COVID-19 infection rates and payment shifts for all cantons. Cantons with an infection rate above 300 per 100,000 residents are marked in red. Excluding these eight cantons, which are mostly located in the southwestern part of Switzerland (the only exception is the urban canton of Basel-Stadt), this leaves us with a near-zero correlation (red trend line). As the lockdown was successful, infection rate growth came to a halt by the end of the lockdown period (third graph in the first column). To summarize, parts of regional shifts in card payments during the lockdown were likely related to infection fear. This was particularly an issue for cantons with very high infection rates.

In Appendix B.3, we look at the correlation with the share of cantonal residents aged 65 and above to mirror infection fear. While we here see opposite (positive) and enduring correlations with excess domestic card-present retail payments, we argue that this could be consistent with infection fear. Elderly people might have increased card payments in order to avoid using cash. However, we must wait for the SNB’s 2020 Survey on Payment Methods for possible confirmation of this view.

The supply side was certainly affected by the lockdown (with the closure of all but essential retail stores) and by the measures imposed on physical distancing and hygiene practices that still have to be respected at the time of writing. We proxy such restrictions by the retail lockdown index per canton as calculated by Faber et al. (forthcoming). In addition to their publicly available data, they calculated a lockdown index for the retail sector per canton. The stronger the retail sector is affected by the lockdown, the lower are the excess domestic card-present retail payments (blue). This correlation remains stable when looking at cantons with low infection rates only (red). While the correlation becomes smaller post-lockdown, the remaining closures, physical distancing, and hygiene practices imposed still seem to exert a negative effect on excess payments.

In Appendix B.3, instead of looking at the cantonal retail-sector lockdown indices, we look at the cantonal shares of the labor force working for the essential retail sector compared to the total labor force working for the retail sector as a whole.^13^ Qualitatively, the same result emerges for the lockdown period as for the full sample. Correlations in the subsample of cantons with low infection rates turn positive, but remain insignificant.

Working from home had been a familiar experience for more than one million office workers in Switzerland before the lockdown (gfs.bern, 2020). With COVID-19, however, it became a new experience for another 300,000 office workers. For the first-ever time, all office workers experienced a near-complete switch to working from home. Such numbers can easily cause payment shifts. Similarly, shopping traffic used to occur during the weekend pre-lockdown, particularly on Saturdays, when many people would go shopping to smaller and larger cities, for instance (Brown et al., 2020). It thus does not come as a surprise that excess domestic card-present retail payments and the home office index are negatively correlated. This is also the case for the pre-lockdown period, which likely reflects a secular trend. The negative correlation remains stable when looking at cantons with low infection rates only (red).

Similar findings result when we replace the home office index with other indicators related to working from home, such as the ratio of the net balance of inter-cantonal and cross-border commuters to the number of economically active persons in the respective canton, the ratio of intra-cantonal commuters to the number of economically active persons, or the canton’s degree of urbanization (see Appendix B.3). The latter two proxy indicators confirm that working from home and commuting are sources of regional shifts, also between differently urbanized areas. Intra-cantonal commuters commute from suburban and rural areas to urban areas. All these indicators are proxies for the fact that shopping shifted from the municipalities where consumers work to municipalities where consumers live.

Shopping tourism abroad is a widespread phenomenon in the border cantons and to a lesser extent in the other cantons. Our shopping tourism frequency indicator shows a negative correlation with excess domestic card-present retail payments for all three periods, with the pre-lockdown coefficient being insignificant (blue). While we would expect this for the pre-lockdown period, it comes as a surprise that we also find negative correlations for the lockdown and post-lockdown periods. Pre-lockdown, we may expect growth to have been lower in cantons that exhibit a high frequency of shopping tourism. As borders remained closed during the lockdown and post-lockdown periods, we would expect to find positive correlations during these periods. This is indeed what we find, if we exclude cantons with high infection rates (red). Next to the high infection rates, some of the excluded cantons—such as Basel-Stadt, Geneva, and Ticino—also show comparatively larger values in other proxies, such as the lockdown index and the home office index. These urban cantons are also likely to suffer more from the absence of tourism and business travel. Anecdotal evidence further suggests that consumers in small and urban cantons with a high intensity of shopping tourism may have been shopping in neighboring cantons, as the retail sector’s capacity in their canton may have reached its limit. Together, these factors may distort the picture in relation to cross-border shopping tourism.

If we replace shopping tourism frequency with accessibility-based indicators that consider the travel time to foreign shopping areas by means of individual motor car traffic and public transport (with or without taking connection frequency into account), qualitatively the same results prevail (see Appendix B.3).

The cash withdrawal values and volumes shown in Fig. 4 illustrate that the outbreak of COVID-19 has resulted in reduced cash usage. These statistics likely understate the long-run impact, as January and February are months of low cash withdrawals (which does not necessarily imply low cash usage).^14^ This is relevant, as we take the difference between cash usage in the two last periods and the first period as the indicator of cash usage. We define cash usage as the cantonal share of cash withdrawals in payments at the POS in the retail sector (as measured by domestic card-present retail payments—card-present payments being the most likely substitute for cash—and cash withdrawals). To minimize this distortion as far as possible, we skip the two first weeks of January when building the share used in Fig. 9, as these 2 weeks regularly show a particularly low cash withdrawal value. Clearly, declining cash usage brings about larger excess card payments. Also, the negative correlation remains stable when adjusting for cantons with high infection rates (red).^15^

## Discussion

In line with other literature using card payment data (Andersen et al., 2020; Baker, Farrokhnia, Meyer, Pagel, & Yannelis, 2020; Brown et al., 2020; Carvalho et al., 2020; Farrell et al., 2020), we provide evidence of significant negative aggregate effects on card payments during the lockdown period. Together with Brown et al. (2020), we provide the first evidence of a substantial rebound of card payments in the post-lockdown period with two rounds of easing (the partial, then full, re-opening of shops). We further present evidence of strongly positive excess retail card payments at the end of our sample that are likely related to the growth in e-commerce, the impossibility of shopping tourism, and cash substitution.

In contrast to the previous literature, our evidence is based on acquirer data instead of issuer data. We further provide evidence based on data that combine debit and credit card payments, rather than presenting evidence-based one type of card. On the one hand, acquirer data allow us to provide a perspective from the supply side (merchant or payee) rather than the demand side (consumers or payers). On the other hand, the combined debit and credit card data allow for a more complete picture of card payments in Switzerland. In the Swiss case, having access to combined data is relevant, as Swiss residents are much more debit card-oriented than US consumers, for instance, but nevertheless pay a significant share of payments by means of credit cards (SNB, 2018).

We find significant retail payment shifts across Swiss regions related to the outbreak of COVID-19. This is confirmed by complementary issuer data in Brown et al. (2020). We find pronounced payment shifts taking place from urban to suburban and rural areas, and also pronounced shifts taking place across cantons. We believe these shifts to be related to seven sources and provide novel descriptive evidence. We first analyze sources that can be directly traced using our card payment data: the accelerated growth in e-commerce due to the outbreak of COVID-19 affected regions differently, i.e., strong e-commerce merchants tend to be located in suburban and rural areas and are not equally spread across cantons; the absence of tourists and business travelers also had a bigger impact on urban and rural areas. A similar picture emerges for the cantons.

By means of proxy indicators, we also evaluate sources that we cannot trace directly in domestic card-present retail payments (after having extracted foreign, then card-not-present retail payments). Fear of infection further reduces consumption beyond lockdown restrictions; cantonal differences in the retail sector’s lockdown exposure (closure of shops, social distancing, and hygiene measures) also drive shifts; working from home drives regional shifts insofar as it moves consumption to areas where people live rather than work; while the absence of shopping tourism abroad increases excess retail payments in Switzerland, its effect on cantonal shifts remains ambiguous (new data after the opening of borders will likely resolve this); and cantonal differences in the adoption of non-cash payment instruments result in an overestimation of retail sales and shifts in payments based on card payments alone.

The economy’s ongoing process of digitalization has meant that an increasing share of non-cash payment instruments, the growth in e-commerce, and working from home were all secular trends, even before the outbreak of COVID-19. However, lockdown-related experiences might have intensified these developments. Lockdown measures and related experiences have seemingly increased employers’ acceptance of employees working from home (Rütti, 2020). In a representative survey conducted by gfs.bern AG on behalf of syndicom, both types of “home workers” (people who already worked at home before the COVID-19 crisis and those for whom working from home has been new) reported an overall positive experience with working from home during the lockdown phase (gfs.bern, 2020). Working from home is likely to increase in importance after COVID-19. As shown in this paper, growth in e-commerce has been overwhelming. There was a strong increase during the lockdown phase, followed by a reduction during the post-lockdown phase. However, e-commerce levels at the end of May were still far above pre-lockdown levels. While lower cash usage is not associated per se with regional shifts in card payments, the adoption of non-cash payment instruments has not been equally strong among the cantons. Part of the regional shift may thus be related to heterogeneous cash substitution.

POS sales in urban areas in particular have fallen, and it is uncertain whether this share will fully recover. It will depend, for example, on whether the changes in consumer behavior that were prompted by the lockdown remain fully or partially valid afterwards on account of more employees working for more days from home, and because of a permanent increase in e-commerce. As a consequence, the POS business in urban areas may have to adapt to longer periods of lower sales and ultimately lower margins. This might have repercussions on companies’ demands for commercial space and on the rents for retail locations, as well as their corresponding valuations. If retail payment shifts persist, it is expected that this will have a corresponding effect on employment, commercial spaces, assortment shifts, and tax revenues in affected areas.

As shown by other COVID-19-related papers, the real-time tracking of the economy by means of payment data harbors potential in many respects. Acquirer data as used in this paper provide a deeper insight into sources that shape the supply side of the economy. Brown et al. (2020) work with aggregated issuer data to shed more light on consumption by Swiss residents (the demand side). These data sets are complementary, and their combined availability on a granular level further increases their potential.

Combining payment system data with transaction-level granularity allows for a more complete picture of both the supply side (the merchant perspective) and the demand side (the consumption perspective) of the retail sector. For instance, combining acquirer data with issuer data might enable us to trace the effect of shopping tourism directly and could be enriched by an e-commerce perspective. Additional data, e.g., on emerging mobile payments, would further allow us to analyze retail payment behavior and shifts to other payment instruments (both traditional and digital invoice-based channels). This represents valuable information for central banks, as operators of payment systems, to accommodate the future requirements of the financial industry and to facilitate and secure the settlement of electronic payments.

With regard to the amount of harvestable payment data in Switzerland, our analysis merely marks the tip of the iceberg. Further payment data are electronically available and await productive analysis. The cost-benefit analysis of acquiring such data sets seems favorable; electronically available data are easily accessible and will become easier to manage and store over time. These data sets might also supplement existing statistical survey data over time, and allow us to shed light on issues for which no other data sources exist (such as shopping tourism).

This research started as a policy project that was meant to foster the real-time tracking of developments in the Swiss economy. Payment data have the advantage of directly measuring economic activity, since payment data are based on completed transactions. In contrast, many other real-time indicators of economic activity are indirect, such as web searches or mobility measurement near points of sale. These indicators measure the intention to make a transaction, but not whether the transaction was ultimately completed or not. Just like other indicators of value added, payment data should not be used unprocessed for economic analysis, but can enable a timely, improved understanding of economic forces, and can serve to inform monetary policy decisions. The journey with real-time payment data has just begun, and empirical research using these data has an exciting and challenging road ahead. Furthermore, we believe it is fair to say that a central bank’s data strategy should necessarily extend to payment data.

### Appendix 2: Additional material

#### Appendix B.1

Figure 12 plots weekly measures of heterogeneity of excess retail card payments among cantons. First, we look at the interquartile range, then we plot the standard deviation of excess retail card payments across cantons. Again, we do so for the value and volume. Clearly, heterogeneity started to increase before the lockdown, and further increased with the lockdown. Interestingly, heterogeneity remained high post-lockdown until the end of May. While excess retail payments recovered and moved into positive territory during the post-lockdown period (see Fig. 1), both measures of heterogeneity have remained close to the elevated levels reached during the lockdown period.

Fig. 12 Weekly measures of heterogeneity of excess retail card payments among cantons. Source: Own calculations, Worldline/SPS

#### Appendix B.2

**Table 2:** Correlations of excess domestic card-present retail payments with selected indicators across pre-lockdown, lockdown, and post-lockdown periods. Source: Own calculations, Worldline/SPS, SFSO

	January/February weeks		Lockdown weeks		April/May weeks	
	Correlation	*p* value	Correlation	*p* value	Correlation	*p* value
**All 26 cantons**						
65 plus population (as % of total)	− 0.01	0.92	0.16	0.07	0.40	0
Cantonal essentials’ share in retail sector	0.16	0.06	− 0.26	0	− 0.29	0
Inter-cantonal and cross-border commuters	− 0.03	0.70	− 0.57	0	− 0.67	0
Intra-cantonal commuters	0.23	0.01	− 0.67	0	− 0.54	0
Urbanization (in % of total communities)	− 0.18	0.04	− 0.55	0	− 0.62	0
Cross-border shopping index, accessibility with own car	− 0.02	0.81	− 0.20	0.02	− 0.23	0.01
**Excluding eight cantons with high COVID-19 infection rates**						
65 plus population (in % of total)	− 0.06	0.59	0.27	0.01	0.47	0
Cantonal essentials’ share in retail sector	0.03	0.77	0.02	0.87	− 0.03	0.78
Inter-cantonal and cross-border commuters	− 0.32	0	− 0.39	0	− 0.59	0
Intra-cantonal commuters	0.04	0.68	− 0.62	0	− 0.57	0
Urbanization (in % of total communities)	− 0.32	0	− 0.49	0	− 0.49	0
Cross-border shopping index, accessibility with own car	− 0.20	0.07	0.19	0.07	0.33	0

#### Appendix B.3

We conduct two robustness checks, acknowledging that we are here talking about very crude robustness checks. The first looks at COVID-19 outliers and whether these cantons bias retail card payments in some way (blue versus red correlations; full sample in blue, no cantons with infection rates above 300 in red). The second robustness check targets the variables. In Fig. 9, we use infection rate, lockdown index for the retail sector, home office index, shopping tourism frequency, and cash usage as proxies. In Fig. 10, we use the share of residents above the age of 65 as an indicator of infection risk, the share of the essential retail sector instead of the lockdown index, the net balance of inter-cantonal and cross-border commuters, intra-cantonal commuters, and the degree of urbanization instead of the home office index, and one shopping tourism accessibility index (motorized individual travel by car) instead of shopping tourism frequency (note that accessibility indices are highly correlated).

Interestingly, a higher share of residents above the age of 65 translates into larger excess domestic card-present retail payments. In interpreting this result, we should consider that card payments do not reflect consumption. Also, one should acknowledge that fewer card payments are observed with increasing age (SNB, 2018). However, cash payments have been widely perceived to be a potential medium of infection and were discouraged by pharmacies and grocery stores. Excess retail card payments should thus be expected to be larger in cantons with a larger share of residents aged 65 and above. The correlations found confirm this. Elderly residents either used cards more often or outsourced shopping to younger people, who in turn use cards more often for payment.

Other findings are in line with the correlations found for the original proxies and are briefly discussed in the main body of this paper.

## Figures and Tables

**Fig. 11:**
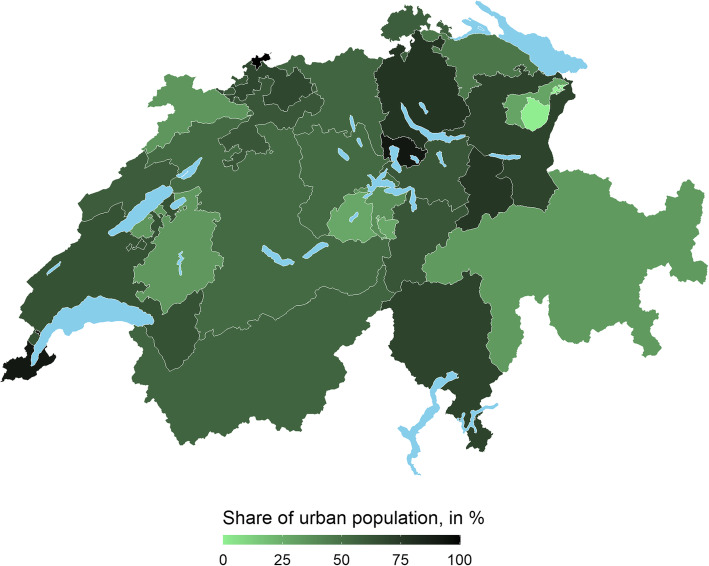
Swiss cantons and their urbanization degrees. Measured as the percentage of residents living in urban municipalities. Source: Own calculations, SFSO

## Data Availability

Data sources are explained in Appendix 1. Other than proprietary data, all data are publicly available.

## Appendix 1: Data and data sources

### Proprietary data

*Debit and credit card transaction data* – Source: SPS Worldline, SNB. Calculations: authors.

Data are available on a transaction level. Payment characteristics and construction of subsets of the data are described in detail in the main body of the text. From this basic data set, we extracted the following variables:

1. *Excess total card payments*
  Excess total card payments are the 2020 aggregated daily value of card payments minus the 2019 aggregated daily value of card payments. Days are matched according to the description in the main body of the text.
2. *Excess retail card payments*
  The same calculation, based on NOGA-47 payments only.
3. *Excess non-retail card payments*
  The same calculation, based on all but NOGA-47 payments.
4. *Excess foreign retail card payments*
  The same calculation, based on payments with foreign card issuer origin only.
5. *Excess domestic card-not-present retail payments*
  The same calculation, only based on payments with domestic card issuer origin that were initiated at the POS site.

*Cash usage* – Source: Brown et al. (2020), SPS Wordline and SNB. Calculations: authors.

Cash usage is constructed as the share of domestic cash withdrawals in payments at domestic POS and domestic cash withdrawals. Daily cantonal cash withdrawal data are taken from Brown et al. (2020); see under “Public data” below. They use SIX BBS AG data, the largest ATM service provider in Switzerland (covering roughly 6000 out of 7000 ATMs; the remaining ATMs are overwhelmingly serviced by Postfinance AG). Daily cash withdrawal data used stems from 4000 ATMs. These ATMs were migrated to the new software before 2020. Data provided by Brown et al. (2020) allow us to consider withdrawals by means of domestically issued bank cards and general-purpose debit cards (but no withdrawals by credit and e-money cards). By the end of our sample, an additional 1000 ATMs had been migrated, with 400 ATMs still waiting for migration. Before the migration (which started in 2018), only withdrawals were reported for which the ATM provider bank and the customer’s card issuer were different. While the software change allows for a more complete picture of cash withdrawals, these data are distorted from 2018 onwards. To get rid of distortions related to this migration, Brown et al. (2020) extract withdrawal data from the beginning of 2020 based on ATMs that were migrated by the end of 2019. They provide aggregate and cantonal data.

The denominator is domestic card-present retail payments, which are probably the closest substitute for cash payments. Brown et al. (2020) build a similar share, using cash withdrawals as the nominator and cash withdrawals plus debit card payments as the denominator. We use POS debit and credit card payments plus cash withdrawals as the denominator. The findings are qualitatively similar.

Our proxy is an indicator; cash withdrawals do not represent cash payments one-to-one. Furthermore, it is important to note the following qualifications. First, cash usage overestimates the share of cash payments, as some consumption has moved from shops to e-commerce. Secondly, it underestimates cash payments because January and February are low-cash withdrawal months due to Christmas and end-of-year effects. Excess cash accumulated at the end of 1 year is spent during the first 2 months of the next year. Thirdly, during times of crisis, people often accumulate extra cash. This would lead to an overestimation of cash usage, as cash is used to store value rather than as a payment instrument. Furthermore, changes in cash withdrawal statistics hinder us from comparing numbers from 2019 with those of 2020, which is why we have to rely on data from a subsample of ATMs to have a time-consistent indicator. However, qualitatively similar results are obtained if data from the full set of ATMs are used. Fourthly, cash withdrawals do not consider cash withdrawals by domestic credit and foreign debit and credit cards, as the dataset by Brown et al. (2020) is limited to withdrawals by domestically issued debit and bank cards. Cash usage by foreign tourists and business travelers might certainly be a potential source of a rebound in cash usage in the future. However, as monthly aggregate SNB payment statistics show, withdrawals by these cards are low and almost vanished after the outbreak of COVID-19. Hence, adding data on withdrawals by foreign-issued cards would lead to a further, biased underestimation of cash withdrawals relative to our current version. The version at hand provides a consistent picture, because both nominator and denominator focus on domestic withdrawals and payments in Swiss francs. Fifthly, ignoring withdrawals by domestic cards other than debit and bank cards underestimates the level of cash withdrawals. However, we desire a relative measure of cash usage. This measure could be biased if there are drastic changes in withdrawals for other cards relative to debit cards, though this is unlikely to be the case because their share is negligible compared to the share of bank and debit card withdrawals (see the monthly statistics on overall payment card transactions on the data portal of the SNB, https://data.snb.ch/en/).

*Shopping tourism frequency –* Source: WEMF AG. Calculations: Demo SCOPE, authors.

We calculate an annual per capita shopping tourism frequency indicator per canton that is based on the MACH consumer survey conducted by Demo SCOPE AG and LINK Marketing Services AG on behalf of WEMF AG. A survey question explicitly asks about the frequency of shopping tourism trips to neighboring countries. Interviewees can answer “each day or several days a week,” “once a week,” “once to three times a month,” “less often,” or “never.” This information is aggregated into an average cantonal annual per capita frequency. We assign a value of 156 shopping trips annually (3 times each week) to the first answer, 52 to the second answer, 24 to the third answer, 4 to the fourth answer, and zero to the last answer. The surveys used encompass the 2018 and 2019 surveys, with more than 9000 interviews. Despite the large number of interviews, the number of answers is slightly below 50 for five cantons. For one canton, the number of answers is below 100. The SFSO notes that extrapolation in these cases is feasible. However, excess domestic card-present retail payments’ correlation with this indicator should be interpreted cautiously.

We do not expect the two survey years to impact the indicator negatively. During these 2 years, the exchange rate EUR-CHF was relatively stable. Also, no policy changes took place that would impact shopping tourism (such as changes in value-added taxes).

*Shopping tourism accessibility* – Source: BAK Economics. Calculations: BAK Economics, authors.

We construct three further indicators for shopping tourism based on the idea of *shopping tourism accessibility*. To do so, BAK Economics applied the BAK Economics Regional Accessibility Model (RAM; BAK Economics, 2019). RAM allows us to construct an indicator that aims to reflect the accessibility of foreign shopping destinations, taking travel times, and the attractiveness of the shopping destinations into account.

The BAK Regional Accessibility Model (RAM) is an economic accessibility model. It calculates the sum of the attractiveness of all outbound destinations (considering GDP as the economic attractiveness factor), multiplied by an accessibility value. The accessibility value exponentially discounts the attractiveness of destinations with travel time (considering the shortest possible travel time). The RAM covers all Swiss municipalities (as sources and destinations) as well as neighboring international regions (as destination only, and at municipality or higher administrative levels) and takes into account motorized individual travel by car (MIV) and public transport (ÖV; with and without consideration of frequency). For more information on RAM, see BAK Economics (2019).

Several deviations from the parameters usually applied in RAM were necessary to reflect the accessibility to foreign shopping destinations. All Swiss destinations were assigned an attractiveness of zero. For neighboring foreign destinations, RAM uses GDP as an indicator for attractiveness. Similarly, to reflect shopping attractiveness, it assumes that a higher level of economic activity in a region is suitable as a proxy for the attractiveness of shopping opportunities. To reflect the neighboring countries’ shopping attractiveness, the following three changes were made to the economic attractiveness values. First, the GDP was re-weighted with the national relative price levels (inverse) of POS shopping-relevant goods (product groups “Nahrungsmittel und alkoholfreie Getränke,” “Alkoholische Getränke, Tabakwaren und Narkotika,” “Bekleidung und Schuhe,” and “Innenausstattung, Ausrüstungsgegenstände und Haushaltsführung”; the source for relative price levels and weights of product groups is the Swiss Federal Statistical Office (SFSO; https://www.bfs.admin.ch/bfs/en/home/).

Secondly, due to the administrative organization of the different countries, a few of the foreign regions included in the RAM are comparatively large and concentrate the GDP of this large area in one destination point. To avoid distortions (as economic activity spread over a wide area does not well reflect the shopping attractiveness of the center), a cap is used. This cap is defined as a maximum value allowed for and is set as equal to 3% of the sum of the attractiveness of all destinations. This limits the attractiveness of 7 out of 551 neighboring foreign destinations covered in the model.

Thirdly, accessibility is defined as a discount function for the attractiveness of a destination and takes into account travel time *x* and a parameter *β* as follows: *exp*(*βx*). The parameter *β* is set to result in a halving of the attractiveness of the destination for every additional 20 min of travel time (which differs from the original RAM). The aggregation on the level of cantons is calculated as the GDP-weighted average of all municipalities.

### Public data:

*Infection fear: COVID-19 infections per 100,000 inhabitants* – Source: www.corona-data.ch.

*Infection fear: ratio of the number of residents above 65 to the total number of residents* – Source: SFSO. Calculations: authors.

*Lockdown index* – Source: Faber et al. (forthcoming). Calculations: Faber et al. (forthcoming); available online: https://wwz.unibas.ch/de/appliedeconometrics/coronavirus/.

We proxy the strength of lockdown measures using the *adjusted lockdown index* per sector by Faber et al. (forthcoming). Their lockdown index measures the physical distance given in all occupations and thus reflects both the restrictions during the lockdown and also how difficult it will be to adhere to hygiene rules after the lockdown. Their base index applies this principle to all occupations, taking into consideration the fact that essential sectors remain open to the public.^16^ We use the adjusted lockdown index that takes into account sources that ease lockdown pressure, such as public sector employment (which suffers least from short-time working or job cuts) and that additionally accounts for the occupations of cross-border commuters.^17^ While the former adjustment is likely not relevant for our application, the latter matters, see Faber et al. (forthcoming) for further information. Additionally, we apply the retail lockdown index per canton that is not provided publicly but can be requested from Faber et al. (2020). Note that the retail lockdown index suffers from a low number of observations. For some cantons, the number of observations on which the index is based lies between 5 and 50. The SFSO notes that extrapolation in these cases is feasible. Twelve out of 26 cantons count observations below 50. However, the correlation between excess domestic card-present retail payments and this indicator should be interpreted cautiously, see Faber et al. (2020) for further information.

*Share of the essential retail sector* – Source: Faber et al. (2020). Calculations: Faber et al. (2020).

The share of the essential retail sector measures the share of the labor force working in the essential retail sector, relative to the labor force working for the whole retail sector. For some cantons, the number of observations on which the index is based lies between 5 and 50. The SFSO notes that extrapolation in these cases is feasible. Twelve out 26 cantons count observations below 50. The correlation between excess domestic card-present retail payments and this indicator should thus be interpreted cautiously, see Faber et al. (2020) for further information.

*Home office index* – Source: Faber et al. (2020). Calculations: Faber et al. (2020), available online: https://wwz.unibas.ch/de/appliedeconometrics/coronavirus/.

The home office index calculates the likelihood that a job can be done effectively from home. Faber et al. (2020) apply the same method as Dingel and Neiman (2020), see Faber et al. (2020) for further information.

*Commuter indicators: net balance of inter-cantonal, cross-border, and intra-cantonal commuters, divided by economically active persons per canton* – Source: SFSO. Calculations: authors.

In terms of commuter statistics, we consider the *net balance of inter-cantonal commuters* weighted by a canton’s economically active persons, the extent of *intra-cantonal commuters* weighted by a canton’s economically active persons, and the *net balance of cross-border commuters* weighted by a canton’s number of economically active persons (based on the most recent statistics available from 2018). The first and second indicators are primarily associated with cantonal shifts in excess retail payments, whereas the third is primarily associated with shifts in excess retail payments among regions with a different degree of urbanization. We therefore consider the total net balance of cantonal commuters when analyzing cantonal shifts, i.e., the sum of net balances of inter-cantonal and cross-border commuters.

*Urbanization degree: percentage of inhabitants living in urban areas* – Source: SFSO. Calculations: authors.

A canton’s *urbanization degree* is measured by the percentage of the population living in urban areas as defined at the level of municipality, see Fig. 11 that illustrates the cantons’ varying degree of urbanization.

*Cantonal cash withdrawal* – Source: SIX BBS AG, Brown et al. (2020). Calculation: Brown et al. (2020); available online: https://public.tableau.com/profile/monitoringconsumptionswitzerland#!/.

Available data contains daily cash withdrawals by domestically issued bank and debit cards. These data are also provided per canton.

## Abbreviations

POS: Point of sale
SFSO: Swiss Federal Statistical Office
ATM: Automated teller machine
RAM: Regional Accessibility Model by BAK Economics
NOGA: “Nomenclature Générale des Activités économiques,” i.e., General Classification of Economic Activities

## Footnotes

1 Worldline/SPS is the largest merchant acquirer in Switzerland. Merchant acquiring is an integral part of card payment transaction processing. Acquirers enable merchants to accept card payments by acting as a link between issuers and payment networks; they provide the infrastructure for card payments (e.g., payment terminals) and offer merchants authorization, clearing and settlement, dispute management, and other services. In contrast, a card issuer provides cards and related services to end users such as consumers.

2 See https://www.bfs.admin.ch/bfs/en/home/statistics/industry-services/nomenclatures/noga.html.

3 We classify the degree of urbanization according to the Swiss Federal Statistics Office “Raumgliederungen” definition of areas with urban character, looking at predominantly urban, suburban, or rural municipalities. We calculate the degree of urbanization of a canton as the share of people living in urban areas.

4 Note that we have data representing payments to merchants located in Switzerland only. Hence, our data mainly reflect domestic transactions by Swiss residents. However, the data do not represent consumption by Swiss residents. For instance, an exception is card-not-present payments by Swiss residents to a foreign merchant. In 2019, card-not-present purchases conducted by cards issued by domestic issuers constituted a non-negligible share of CHF 11,621 million or 11.6% of total purchases conducted with domestic debit and credit cards (see SNB data portal on payments and cash withdrawals for more information). Also, the data do not tell us anything about consumption by Swiss residents abroad, such as tourism expenditure and expenditure related to shopping tourism. For this, we would need card issuer data instead of acquirer data. Furthermore, acquirer data also include payments by foreign-issued cards. These payments originate by foreign tourists and business travellers and foreign consumers’ expenditures at Swiss e-commerce merchants.

5 We associate payments with cards from domestic issuer as payments by Swiss residents. In contrast, we associate payments with cards by foreign issuer as payments by foreign countries’ residents.

6 The data does not cover card payments at merchants acquired by PostFinance and other acquirers. We calculate covered card transactions by comparing our single acquirer data to aggregate issuer data (monthly statistics on overall payment card transactions are found on the data portal of the SNB, https://data.snb.ch/en/). Acquirer data exclusively cover transactions settled in Switzerland for holders of cards issued by both domestic and foreign issuers. Issuer data exclusively cover transactions by holders of cards issued by domestic issuers only, regardless of the payee’s origin (foreign or domestic). We compare with the 2019 shares of domestically issued cards used for transactions with domestic merchants. In 2019, the data cover around two thirds of the total value spent, compared to all card payments.

7 Lockdown regulations in Switzerland were relatively loose; for instance, there was no nation-wide curfew was imposed in other countries.

8 NB: borders and sports and entertainment venues remained closed until mid-June.

9 We chose this version over normal weeks, as the lockdown had a more drastic impact than the subsequent easing steps. The two easing steps included took place on Mondays. However, their effects were more gradual than the lockdown effect. This matters only for weekly comparisons. The exact dates are used when comparing longer periods.

10 One should keep in mind that the Easter weekend, with Good Friday and Easter Monday, is a public holiday in Switzerland. It began on Friday, April 19 in 2019, and on Friday, April 10 in 2020. This matters for the direct comparison of these two particular weeks but does not impair the comparison of the whole lockdown period with the corresponding period in 2019.

11 It remains unproven whether or not cash is a fomite, i.e., whether it can transfer pathogens. See also the WHO controversy on the issue: https://www.marketwatch.com/story/who-we-did-not-say-that-cash-was-transmitting-coronavirus-2020-03-06.

12 Note that we associate POS sales with cash and card-present payments and e-commerce sales with card-not-present payments.

13 The essential retail sector is defined as merchants selling essential goods such as food, drugs, and gas.

14 See SNB statistics on payments and cash withdrawals: https://data.snb.ch/en/topics/finma#!/cube/zavezaluba.

15 There is no alternative proxy indicator to discuss for cash usage.
