# Supplementary figures and images for: COVID-19 and regional shifts in Swiss retail payments
Source: Swiss J Econ Stat. 2020 Sep 25;156(1):14. doi: 10.1186/s41937-020-00061-x (PMC8091136; doi:10.1186/s41937-020-00061-x)

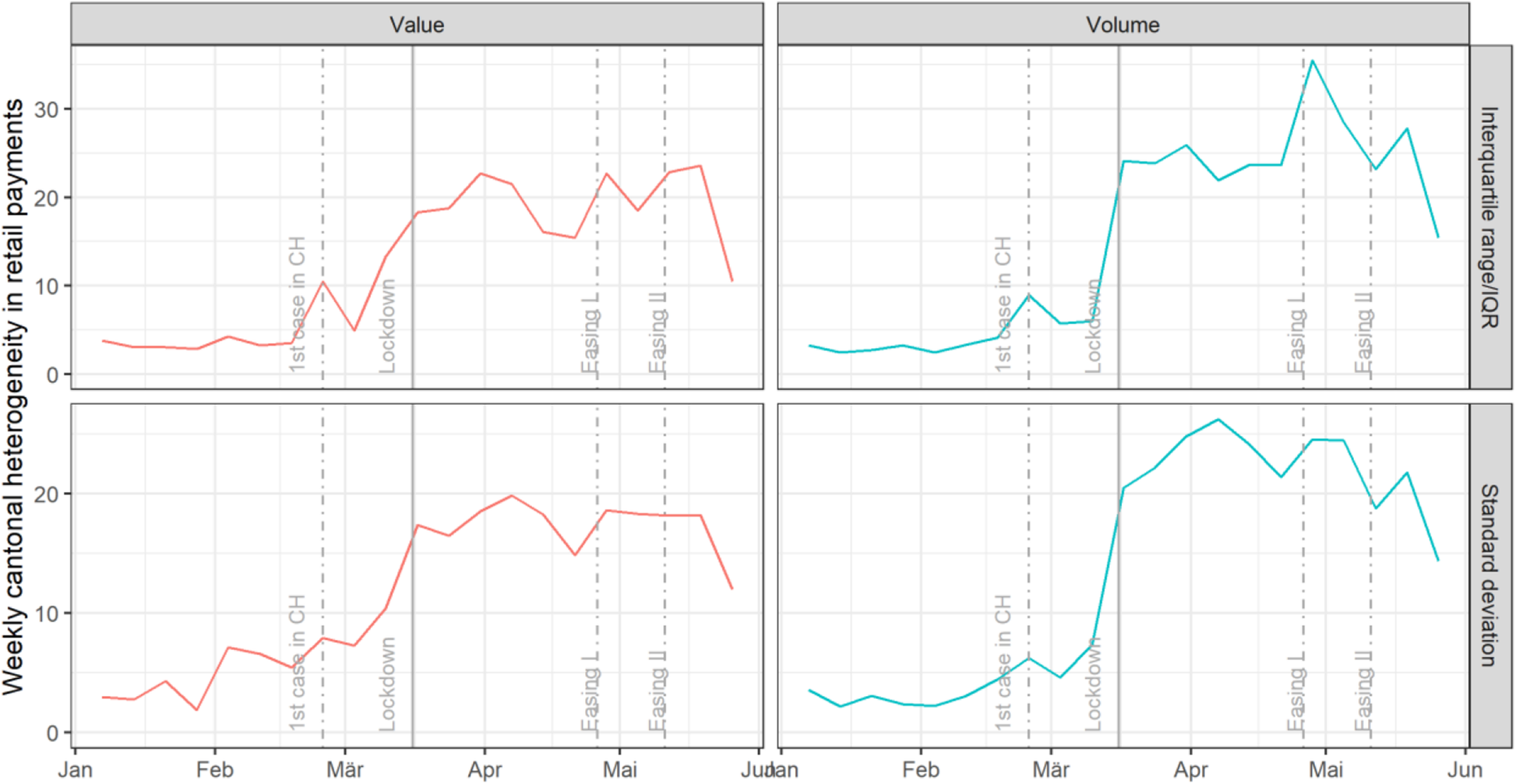

Supplement: Supplementary file 1 — Weekly measures of heterogeneity of excess retail card payments among cantons. Source: Own calculations, Worldline/SPS [file 41937_2020_61_Fig12_HTML.png]
